# Soluble Flt-1 and PlGF: New Markers of Early Pregnancy Loss?

**DOI:** 10.1371/journal.pone.0018041

**Published:** 2011-03-23

**Authors:** Shanthi Muttukrishna, Michelle Swer, Sangeeta Suri, Amna Jamil, Jean Calleja-Agius, Subrata Gangooly, Helen Ludlow, Davor Jurkovic, Eric Jauniaux

**Affiliations:** 1 Department of Obstetrics and Gynaecology, Anu Research Centre, University College Cork, Cork University Maternity Hospital, Wilton, Cork, Republic of Ireland; 2 University College London Institute for Women's Health, University College London, London, United Kingdom; Indiana University, United States of America

## Abstract

Recent data have indicated a relationship between placental oxygen and angiogenic protein levels in the first trimester of normal pregnancies. Our objective was to investigate if maternal serum levels of angiogenic factors Soluble vascular endothelial growth factor (VEGF) receptor 1 (sFlt-1), soluble Endoglin and placental growth factor (PlGF) are altered in women with symptoms of threatened miscarriage (TM) and if they are predictive of a subsequent miscarriage. Blood samples were collected at 6–10 weeks from women presenting with TM (n = 40), from asymptomatic controls (n = 32) and from non- pregnant women in their luteal phase (n = 14). All samples were assayed for serum level of sFLT-1, PlGF, sEndoglin and HSP70 using commercial ELISAs. Samples were analysed retrospectively on the basis of pregnancy outcome. TM group included 21 women with a normal pregnancy outcome and 19 with subsequent complete miscarriage. The latter subgroup had significantly lower mean maternal serum (MS) sFlt-1 (83%, P<0.001) and PlGF (44%, P<0.001) compared to those with a normal pregnancy outcome. Asymptomatic control pregnant women had similar MS levels of sFlt-1 and PlGF compared to the TM patients with a normal outcome. The mean MS sFlt-1 (>10 fold) and MS PlGF (∼2 fold) levels were significantly (P<0.001) higher in control pregnant women compared to the non-pregnant group in the luteal phase of the menstrual cycle. Soluble Endoglin was not altered in the normal pregnant women compared to non pregnant women, although lower in the TM subgroup with a subsequent miscarriage (∼25%, P<0.001) compared to TM with a live birth. There was no significant difference in the mean MS HSP 70 levels between the different groups. This study shows that sFlt1 and PlGF MS levels are increased by several folds in early pregnancy and that MS sFlt-1 and MS PlGF are markedly decreased in threatened miscarriage patients who subsequently have a miscarriage suggesting these proteins are sensitive predictive markers of subsequent pregnancy loss.

## Introduction

Placenta related disorders of pregnancy are the most common complications of human pregnancies. Collectively, complete miscarriages, missed miscarriages, recurrent miscarriages, threatened miscarriages and placental insufficiency associated or not with maternal hypertensive disorders affect more than 30% of clinical pregnancies in humans. These placental disorders are exceptional in other mammalian species [1], [2].

Human placentation is characterized by the highly invasive nature of the conceptus which embeds itself completely within the maternal uterine endometrium and superficial myometrium and by a remodelling of the tip of the maternal spiral arteries [1]. In normal pregnancies, the earliest stages of development takes place in a low oxygen (O_2_) environment [3], [4]. This physiological hypoxia of the early gestational sac protects the developing fetus against the deleterious and teratogenic effects of O_2_ free radicals. A stable O_2_ gradient between the maternal uterine decidua and the feto-placental tissue is also an important factor in trophoblast differentiation and migration, normal villous development and angiogenesis [5], [6].

Previous studies have shown that in normal pregnancies there is a physiological oxidative stress in the placental tissue at around 9–10 weeks which is evidenced by an increase in HSP70 activity mainly in the periphery of the primitive placenta [7]–[9]. The villous changes observed in the periphery of the placenta during the formation of the fetal membranes are identical to those found in the missed-miscarriage indicating a common mechanism mediated by oxidative stress [8]–[10]. A missed miscarriage is identified before the expulsion of the fetus or placental tissue. It is diagnosed by ultrasound on the basis of absent fetal heart activity after 5 weeks of gestation or in the presence of an empty gestational sac. Threatened miscarriage (TM) is diagnosed when normally grown live fetus is found on ultrasound in the presence of vaginal bleeding [11]. TM is associated with focal oxidative stress in the definitive placenta and increases the possibility of adverse pregnancy outcomes such as miscarriage, preterm delivery and premature rupture of the membranes [11].

The oxidative stress and rise in oxygenation may alter the synthesis of various placental proteins. Maternal serum concentrations of hCG peak towards the end of the first trimester and oxidising conditions promote assembly of the sub-units in vitro [12]. Our recent data showing an association between intrauterine O_2_ concentration in vivo and inhibin A and sFlt-1 concentrations in early pregnancy suggest that specific placental proteins may be regulated by intrauterine O_2_ concentration [13].

In early pregnancy failure, the development of the placento-decidual interface is severely impaired leading to early and widespread onset of maternal blood flowing continuously inside the placenta, together with major oxidative stress induced tissue degeneration [8], [9], [13]. The excessive entry of maternal blood inside the placenta in the early stage of most miscarriages is unrelated to conceptus karyotype^10^. In more than two-thirds of the cases of missed miscarriage, there is anatomical evidence of defective placentation with reduced cytotrophoblast invasion of the endometrium, reduced transformation and incomplete plugging of the spiral arteries [14]–[17].

Angiogenesis is characterised by increased vascular permeability, endothelial cell proliferation and migration. It is regulated by various pro- and anti-angiogenic factors, angiopoietins and matrix metalloproteinases. Anti-angiogenic and pro-angiogenic factors are reported to play an important role in the pathophysiology of pre-eclampsia (PE) [18]. Soluble Endoglin (sEndoglin) is a soluble TGF-beta co-receptor. sEndoglin inhibits the formation of blood vessels and induces vascular permeability and hypertension [19]. sFlt-1 is soluble vascular endothelial growth factor (VEGF) receptor 1. sFlt-1 binds to the proangiogenic growth factors VEGF and placental growth factor (PlGF), thereby neutralizing their functions. Angiogenic growth factors VEGF-A and (PlGF) have been investigated extensively in normal and abnormal placental vascular development [20]–[22]. Recent studies suggest that pre-eclampsia, hypertension and proteinuria may be due to an excess of circulating anti-angiogenic growth factors, most notably sFlt1 and sEndoglin [19]–[21].

Angiogenic factors have not been previously evaluated in early pregnancy complications. Abnormal vascularisation of the placenta with increased oxidative damage is a common aetiology of pre-eclampsia, fetal growth restriction due to placental insufficiency and early pregnancy failure [1], [2]. The aims of this study were to determine whether TM which is associated with a focal oxidative stress in the definitive placenta is also associated with changes in angiogenic factors and to investigate if maternal serum levels of sFlt-1, PlGF and sEndoglin can be potential sensitive markers of a subsequent complete miscarriage in women presenting with a TM in the first trimester of pregnancy.

## Methods

All pregnant women were recruited from the University College London Hospital (UCLH) and the non-pregnant control samples were obtained from UCL staff and students. This study was approved by the University College London Hospitals Committee on the Ethics of Human research. All women gave informed written consent to participate in the study.

TM was defined as vaginal bleeding with or without abdominal pain during the first trimester of pregnancy and an ultrasound examination showing an intrauterine gestational sac with a normally grown live fetus with regular fetal heart beat within normal ranges and with or without a subchorionic hematoma. The blood sample was obtained from patients who attended the early pregnancy unit with symptoms of TM. A viable pregnancy was confirmed by scan on the day of blood collection. The outcome of pregnancy was monitored until term.

### Study groups

Blood samples were collected as part of a prospective cohort study of women attending the early pregnancy clinic during the first trimester of pregnancy. The outcomes of these patients were followed as part of the prospective cohort study. TM with a subsequent miscarriage outcome (n = 19) and TM with a subsequent term live birth outcome (n = 21) were selected retrospectively from singleton spontaneous conceptions matched for gestational age (±5 days) at sampling, maternal age (±5 years), BMI and smoking status, for analysis. For comparison, blood samples collected from asymptomatic pregnant women (n = 32) matched for gestational age (±5 days) at sampling, maternal age (±5 years), BMI and smoking status in the first trimester and non-pregnant women in the luteal phase (not on any medication) were analysed (20–35 years, n = 14). Demographic data of the different groups are summarized in Table 1. Asymptomatic antenatal controls and TM live births had normal pregnancy outcome at term.

**Table 1:** Demographic details of the participants in the study given as median and interquartile range (25^th^, 75^th^ percentiles).

	Non pregnant (Median (25^th^ &75^th^ percentiles) N = 14	Control pregnant N = 32	TM-Live birth N = 21	TM-Miscarriage N = 19
Gestation (days)	-	50.5 (46, 58.5)	50.5 (42, 60)	51 (47, 59)
Maternal Age (years)	26 (24.75, 32.25)	32 (28.75, 35)	31 (25.5, 35)	34 (32, 40)
BMI	22.5 (20.16, 24.5)	22.61 (21.75, 25.52)	23.5 (21, 28.75)	24.5 (21.25, 27)
Birth weight (grams)	-	3621(3123, 4057)	3220 (2738, 3580)	

All blood samples were taken in the first trimester between 6–10 weeks in gestation from Jan 2008-April 2010. Serum was separated and frozen at −80°C until analysis.

### Immunoassays

All samples were assayed for Soluble Flt-1, PlGF, sEndoglin and heat shock protein 70 (HSP70) using commercial ELISAs. Quantakine ELISA kits for PlGF and sFlt-1 and reagents from a duo-set for sEndoglin was used from R&D systems (UK) to measure the respective proteins in serum samples according to the manufacturer's protocol at the Reproductive Sciences laboratory, UCL and at Anu Research Centre, UCC.

The minimum detection limit for the respective assays was PlGF: 3.9 pg/ml, sEng: 62.5 pg/ml, sFlt-1 31.3 pg/ml. Heat shock protein 70 was measured in serum using a highly sensitive assay kit from Assays Design (Cambridge Bioscience, UK). The detection limit of HSP 70 was 0.2 ng/ml. All samples were assayed in duplicates and the intra and inter assay variations were <12% for all assays.

### Statistical Analysis

We carried out non parametric general linear model analysis of variance controlling for maternal age using Dunnett's T3 posthoc tests because data were not normally distributed. Gestation at sampling and BMI were not significantly different among the groups. Correlation analysis was carried out to study the relationship between the parameters. Data was presented as scatter plot with median and interquartile range and P values were considered significant at P<0.05.

## Results

**Figure 1:**
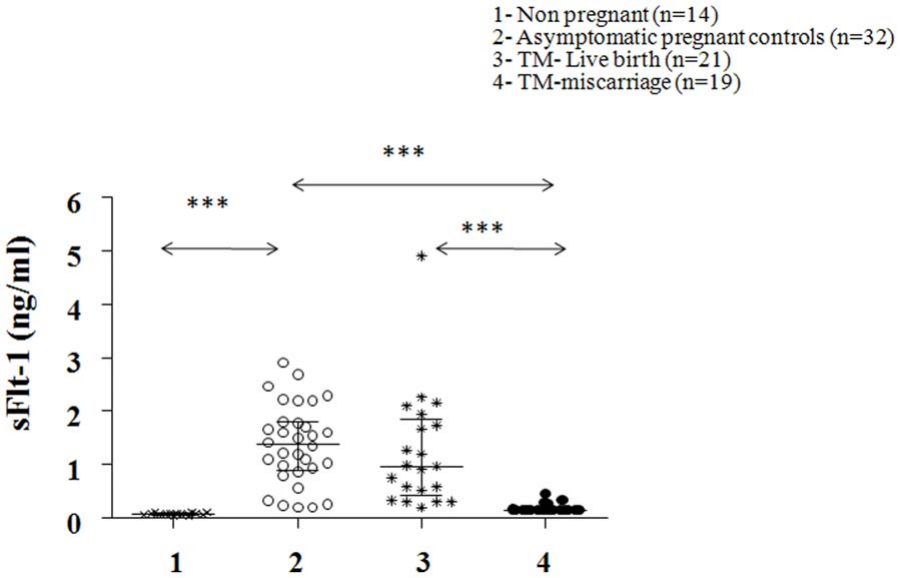
Scatter plot of circulating levels of serum soluble VEGF receptor 1 (sFLT-1) in the different groups of women with median and interquartile range. Group 1 (non-pregnant women, n = 14), group 2 (normal pregnant women, n = 32), group 3 (threatened miscarriage patients with a live birth outcome, n = 21) and group 4 (threatened miscarriage patients with a subsequent miscarriage, n = 19). General linear analysis of variance was carried out to study the statistical significance between the groups with posthoc tests. P<0.001 = ***.

The mean MS sFlt-1 level was significantly (P<0.001) higher (>10 fold increase) in normal pregnancy compared to the non-pregnant group whereas mean MS sFlt-1 levels were significantly (86% decrease, P<0.001) lower in the TM subgroup with a subsequent miscarriage compared to asymptomatic controls. TM subgroup with a subsequent miscarriage had significantly lower levels of sFlt-1 (83% decrease, P<0.001) compared to TM subgroup with a subsequent live birth (Figure 1).

**Figure 2:**
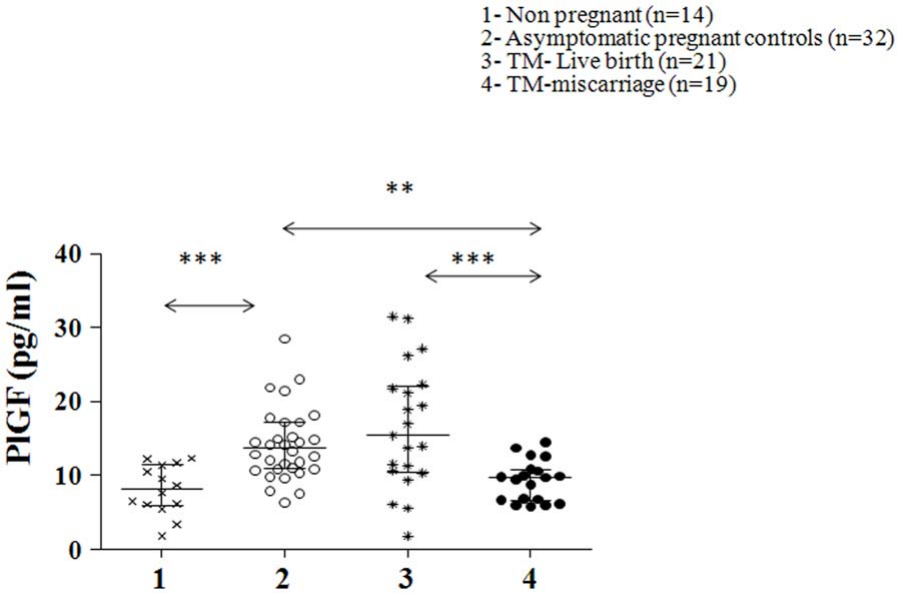
Scatter plot of circulating levels of serum placental growth factor (PlGF) in the different groups of women with median and interquartile range. Group 1 (non-pregnant women, n = 14), group 2 (normal pregnant women, n = 32), group 3 (threatened miscarriage patients with a live birth outcome, n = 21) and group 4 (threatened miscarriage patients with a subsequent miscarriage, n = 19). General linear analysis of variance was carried out to study the statistical significance between the groups with posthoc tests. P<0.001 = ***, P<0.01 = **.

The mean MS PlGF levels were higher in pregnancy than in non-pregnant controls (∼2 fold increase, P<0.001). PlGF levels were lower in TM patients with a subsequent miscarriage (44% decrease, P = 0.001) compared to TM patients who subsequently had a term live birth. TM patients with subsequent miscarriage had lower levels of PlGF (42% decrease, P = 0.002) compared to asymptomatic pregnant women (Figure 2).

**Figure 3:**
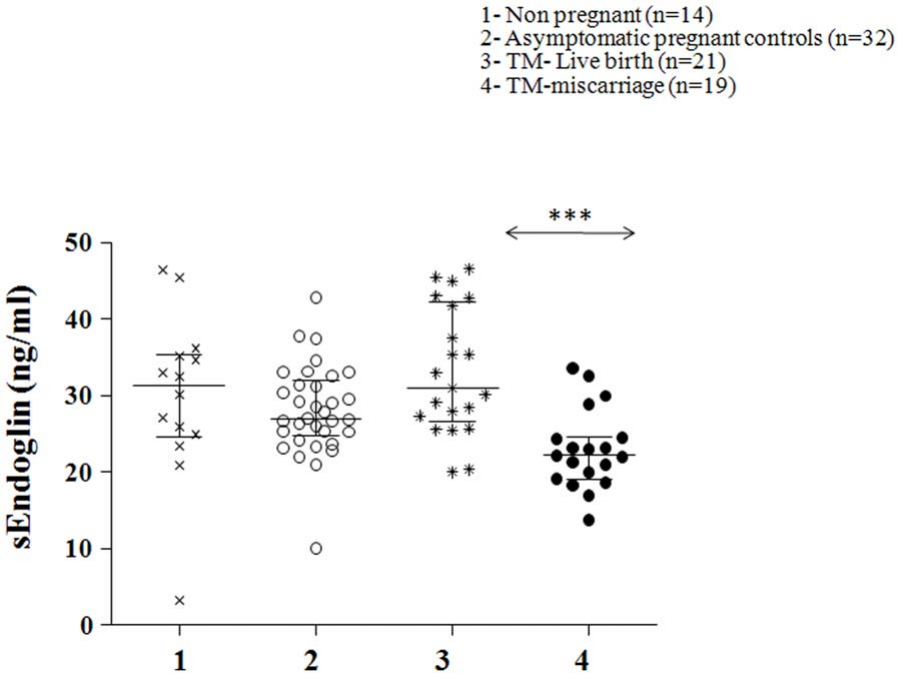
Scatter plot of circulating levels of serum soluble Endoglin (sEndog) in the different groups of women with median and interquartile range. Group 1 (non-pregnant women, n = 14), group 2 (normal pregnant women, women, n = 32), group 3 (threatened miscarriage patients with a live birth outcome, n = 21) and group 4 (threatened miscarriage patients with a subsequent miscarriage, n = 19). General linear analysis of variance was carried out to study the statistical significance between the groups with posthoc tests. P<0.001 = ***.

**Figure 4:**
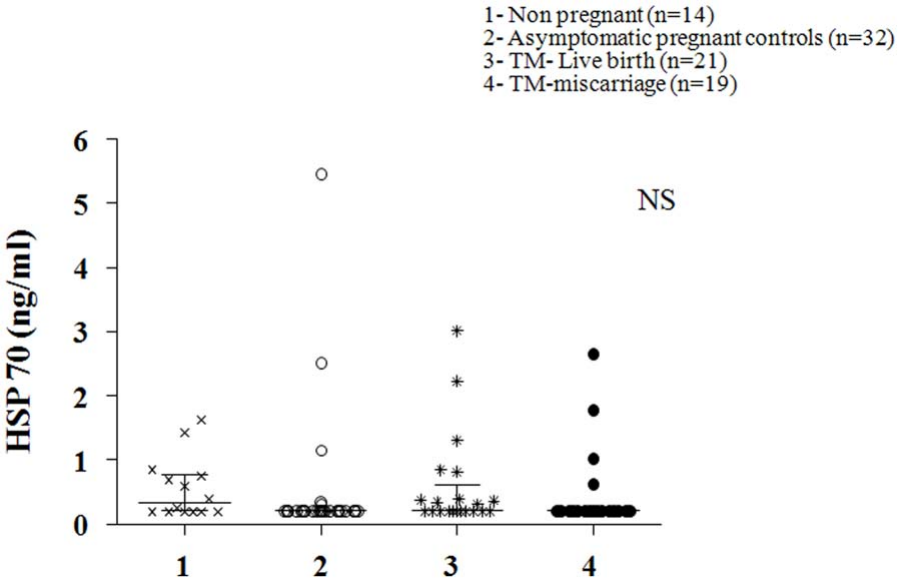
Scatter plot of circulating levels of serum heat shock protein 70 (Hsp 70) in the different groups of women with median and interquartile range. Group 1 (non-pregnant women, n = 14), group 2 (normal pregnant women, n = 32), group 3 (threatened miscarriage patients with a live birth outcome, n = 21) and group 4 (threatened miscarriage patients with a subsequent miscarriage, n = 19). General linear analysis of variance was carried out to study the statistical significance between the groups with posthoc tests. NS = not significant.

There was no significant difference in the mean sEndoglin levels between the non-pregnant and control pregnant women. However, sEndoglin levels were modestly lower (∼25%, P<0.001) in the TM women who had a miscarriage compared to TM women who had a live birth (Figure 3). Mean MS HSP70 levels were not significantly different between all groups of pregnant women and non-pregnant controls (Figure 4).

Bivariate correlation analysis showed a positive correlation between gestational age and MS sFlt-1 (r = 0.5, P<0.001) and PlGF (r = 0.42, P<0.001). Serum levels of sFlt-1 and PlGF (r = 0.57, P<0.001) were significantly associated. Endoglin was positively correlated with gestational age (r = 0.284, P = 0.012).

## Discussion

This study shows that maternal levels of sFlt-1 (83%) and PlGF (44%) are distinctly lower in TM complicated by subsequent miscarriage compared to normal pregnant controls and TM with subsequent live birth. Our findings suggest that these molecules could be used as potential predictive markers of miscarriage in these women presenting with TM during the first trimester of pregnancy at 6–10 weeks gestation. The development of algorithms to predict miscarriage could help triaging for high-risk couples with a history of recurrent miscarriages and for women presenting with bleeding in early pregnancy. MS PlGF and MS sFlt-1 levels were similar in TM with a normal obstetric outcome and asymptomatic controls suggesting that the pathophysiology of TM i.e. development of a subchorionic bleed does not alter the production of the proteins by the placenta in early pregnancy.

Previous studies have shown that endocrinological parameters such as hCG, progesterone [23], [24], PAPP- A and inhibin A [11] levels are decreased in the first trimester in patients with symptoms of TM who subsequently have a complete miscarriage compared to those with a normal obstetric outcome. A recent community based cohort study has investigated the association of self reported first trimester bleeding with a subsequent miscarriage [25]. The authors concluded that patients with heavy bleeding and associated pain in the first trimester have three times more risk of a complete miscarriage compared to asymptomatic women.

In the recent years, pro- and anti-angiogenic proteins have been reported to be useful biomarkers in predicting pregnancy complications such as pre-eclampsia and fetal growth restriction [19]–[21]. In this study, we found that the levels of sFlt-1 rose >10 fold in pregnancy at 6–10 weeks gestation compared to luteal phase of the menstrual cycle confirming that the feto-placental unit is a major source for this molecule in early pregnancy. P1GF also increased by almost 2 fold in early pregnancy. By contrast, no difference was found in the concentrations of sEndoglin between samples from non-pregnant women and from early pregnancy controls suggesting that there could be multiple sources of this protein.

Independent of the cause of an early pregnancy failure, the premature and excessive entry of maternal blood inside the placenta has two effects on the villous tissue. First a direct mechanical effect with most of the villi becoming progressively embedded inside large intervillous blood thrombi and secondly an indirect widespread O_2_-mediated effect causing oxidative damage leading to major apoptosis and necrosis of the villous trophoblast [8], [26]. Overall, the consequences are placental degeneration with complete loss of syncytiotrophoblast function and detachment of the placenta from the uterine wall. This mechanism is common to all miscarriages irrespective of the time at which it occurs in the first trimester depending on the aetiology [1]. Oxidative stress is also involved in the development of other placental-related disorders such as pre-eclampsia and premature rupture of the placental membranes [8], [9].

We have recently shown that O_2_ concentration in the placental bed blood is inversely related to sFlt-1 in early pregnancy [13]. A decreased level of sFlt-1 in MS prior to a complete miscarriage suggests that impaired placentation may be associated with placental metabolic changes before the appearance of clinical symptoms of miscarriage and these changes are modulated by an abnormal increase in O_2_ concentration inside the placenta after implantation.

Overall women presenting with TM are also more likely to deliver prematurely and/or present with preterm pre-labour rupture of membranes [11]. The presence of a haematoma may also be associated with a chronic inflammatory reaction in the decidua. This results in persistent myometrial activity and expulsion of the pregnancy or progressive cellular dysfunction and/or damage to cellular layers of the membranes leading to preterm rupture and delivery [33].

HSP70 is a sensitive marker of oxidative stress in tissues. It is reported to have a peak in the expression of HSP70 in the placental tissue from normal pregnancies at 8–10 weeks when the fetal membranes start to form [3]. We also found that the immunoreactivity for HSP70 is greater in samples from peripheral than from central regions of normal placentas and in the whole placental tissue in missed miscarriage [3]. Early blood flow is restricted to the peripheral regions of most normal placentas whereas in missed miscarriages it is most common in central regions or throughout the placenta [3], [10]. Circulating levels of HSP 70 in pre eclampsia, HELLP syndrome and preterm delivery are reported to be increased in MS compared to normal pregnancy suggesting HSP70 may reflect inflammation and oxidative stress [26]. In the present study, we found no difference in the MS HSP70 among the subgroups suggesting that peripheral circulating HSP70 may not reflect focal changes in intrauterine tissues. HSP70 is therefore unlikely to be useful in the screening for early pregnancy loss.

There is evidence that the circulating cytokine levels and the cytokine profile in the decidua are different in women who experience recurrent miscarriages [27]–[30] but the exact interaction of each of these cytokines with the invading trophoblast is not well defined. Decreased expression of angiogenic factor genes for basic fibroblast growth factor (FGF), intergrin αV and vascular endothelial growth factor (VEGF) has also been observed in placental samples from recurrent miscarriage patients [31]. There are no reliable assays available to measure ‘total’ VEGF in circulation and circulating levels of VEGF are almost below detection in early pregnancy [13]. In the present study, we found significantly (P<0.001) lower levels of sFlt-1 and P1GF in TM patients with a subsequent miscarriage than in TM patients with a subsequent live birth. Whereas the lowering of P1GF can be related to a decreased syncytiotrophoblast synthesis, lower levels of sFlt-1 may be compensatory as the placenta may be producing more VEGF and less sFlt-1 that is bound to VEGF. On the other hand, both VEGF and the receptor production may be lower in patients who subsequently have a miscarriage thus reflecting lower levels of sFlt-1 in maternal circulation in these cases. Although lower PlGF is associated with pre-eclampsia and miscarriage, levels of sFlt-1 and sEndoglin are raised in pre-eclampsia and decreased in miscarriages suggesting a different mechanism in miscarriage.

At present, there is no reliable marker to predict the clinical outcome of women presenting with TM in the first trimester of pregnancy although several potential markers have been studied [23], [24]. We have shown previously that at term, sFlt-1 rapidly declines after the removal of the placenta with a ∼50% decline observed within 1 hr of delivery [32]. This suggests that, with its short half-life in the maternal circulation, sFlt-1 could be a more sensitive marker than molecules such as hCG which have a longer half-life.

In this study, we have demonstrated that sFlt-1 and PlGF could be new sensitive predictors of a subsequent miscarriage in TM patients in the first trimester. Further larger studies are required to investigate the predictive efficiency of these markers.
